# Notch activity in the nervous system: to switch or not switch?

**DOI:** 10.1186/1749-8104-4-36

**Published:** 2009-10-02

**Authors:** Elise Cau, Patrick Blader

**Affiliations:** 1 Université de Toulouse, UPS, Centre de Biologie du Développement (CBD), 118 route de Narbonne, F-31062 Toulouse, France; 2 CNRS, CBD UMR 5547, F-31062 Toulouse, France

## Abstract

The Notch pathway is instrumental for cell fate diversification during development. Pioneer studies conducted in *Drosophila *and more recent work performed in vertebrates have shown that in the nervous system, Notch is reiteratively employed when cells choose between two alternative fates, a process referred to as a binary fate decision. While the early (neural versus epidermal) fate decisions mainly involve an inhibitory effect of Notch on the neural fate, late fate decisions (choice between different subtypes of neural cells) have been proposed to involve a binary switch activity whereby Notch would be instructive for one fate and inhibitory for the other. We re-examine this binary switch model in light of two recent findings made in the vertebrate nervous system. First, in the zebrafish epiphysis, Notch is required to resolve a mixed identity through the inhibition of one specific fate. Second, in the murine telencephalon, Notch regulates the competence of neural progenitors to respond to the JAK/STAT pathway, thereby allowing for the induction of an astrocyte fate. In neither case is Notch instructive for the alternative fate, but rather cooperates with another signalling pathway to coordinate binary fate choices. We also review current knowledge on the molecular cascades acting downstream of Notch in the context of neural subtype diversification, a crucial issue if one is to determine Notch function as an instructive, permissive or inhibitory signal in the various cellular contexts where it is implicated. Finally, we speculate as to how such a 'non-switch' activity could contribute to the expansion of neuronal subtype diversity.

## Notch in the fly nervous system: selection of a neural progenitor and specification of neuronal subtype identity

The Notch pathway is a crucial signalling pathway involved in development and disease that functions through the binding of transmembrane ligands (the DSL proteins, for Delta-Serrate-Lag2) to transmembrane receptors (the Notch molecules) on adjacent cells. Such binding triggers the proteolysis of Notch and the release of its intracellular domain (the so-called Notch-intra fragment), which is translocated into the nucleus. Canonical Notch signalling involves the binding of Notch-intra to DNA-binding cofactors belonging to the CSL family (for CBF1 in human, Suppressor of Hairless in *Drosophila *and Lag-1 in *Caenorhabditis elegans*) [1,2]. Notch-intra/CSL complexes subsequently activate transcription of target genes through the recruitment of the histone-acetyl transferases CBP/p300 [3,4] and PCAF [5].

**Figure 1:**
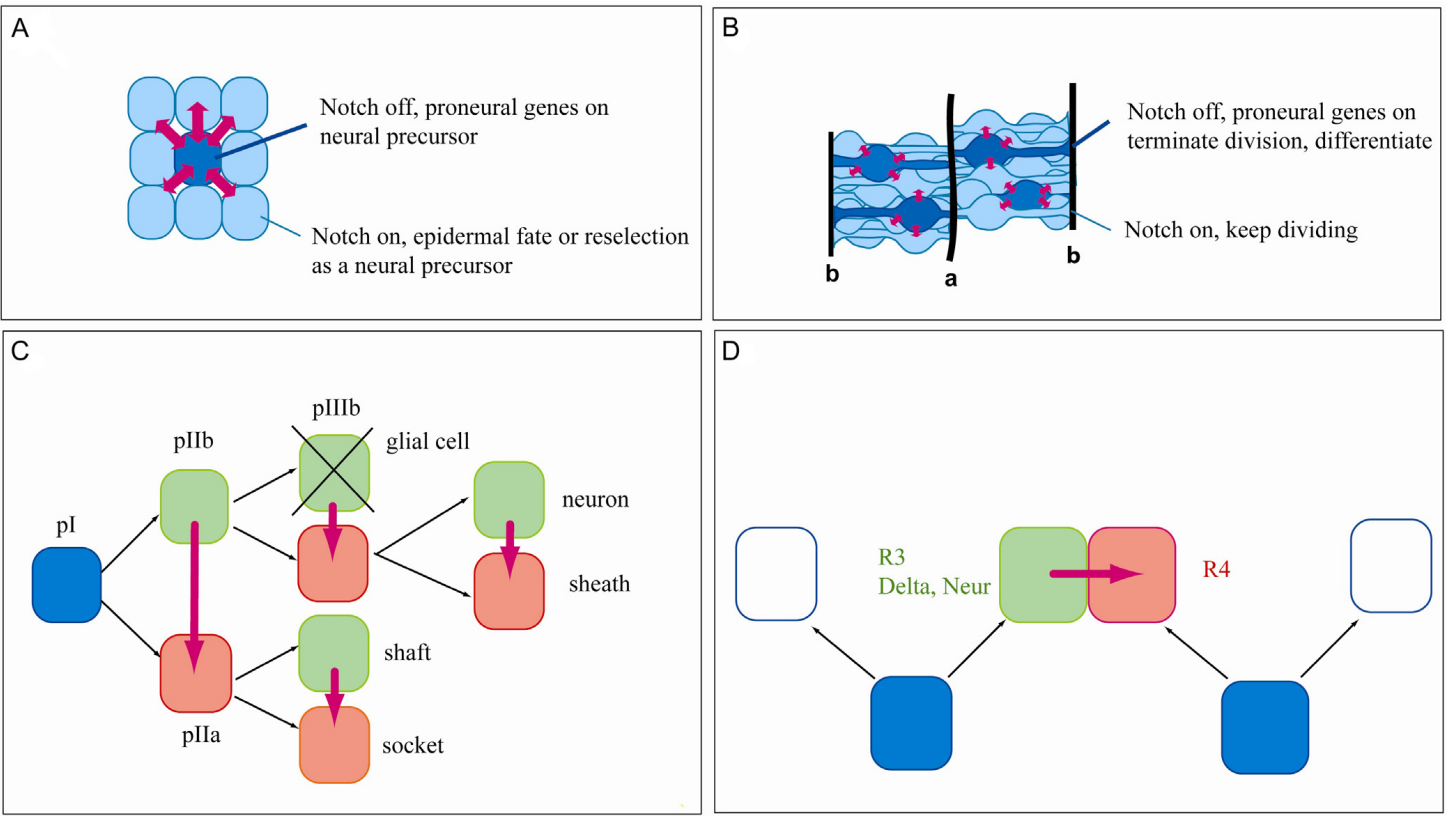
**Roles for Notch during neurogenesis: selection of a neural progenitor and specification of neuronal subtype identity**. (A, B) Notch communication (in pink) is required for the selection of neural progenitors both in *Drosophila *(A) and vertebrates (B). Arrows indicate the directionality of Notch signalling. Note that at the beginning of the process Notch communication is bidirectional. The letters 'a' and 'b' indicate the apical and basal sides of the neural tube. Dark blue, 'Notch off' cell; light blue, 'Notch on' cell. (C, D) Notch is also required during the specification of neural subtypes, which also consists of binary fate decisions but between two different neural subtypes. These binary decisions can either involve sister cells, as is the case during the formation of *Drosophila *sense organs (C) or cells that are not linearly related, as in the case of the R3 and R4 photoreceptors of the *Drosophila *eye (D). (C) Once specified as a neural progenitor, the SOP (Sensory organ precursor; or pI) divides to generate two cells, pIIa and pIIb, which communicate via Notch. Subsequent divisions generate the four cells of the sensory organs as well as a glial cell that will undergo apoptosis [64-66].

Notch activity has been extensively studied in the *Drosophila *nervous system, where it regulates cell fate choice in several different contexts. First, the Notch pathway is required to select single cells to become neural precursors from a cluster of equipotent progenitors that express basic helix-loop-helix (bHLH) transcription factors called proneural genes (Figure 1A). Expression of proneural genes endows cells with a neural potential as these genes are both necessary and sufficient for the formation of neural progenitors [6]. In situations where Notch activity is absent, all the cells from the clusters retain expression of the proneural genes and become neural cells [7-11]. Conversely, when Notch signalling is activated constitutively, all the cells of the cluster acquire an epidermal fate [12-14]. These observations led to the following model whereby cells within proneural clusters communicate via an inhibitory feed-back loop of Notch activity. After several iterations of the loop, only one cell of the cluster downregulates the Notch pathway, retains proneural expression and becomes a neural precursor. The remaining cells, which still have the neural fate inhibited by Notch, will either be reselected during a second wave of neurogenesis or secondarily adopt an epidermal fate [15]. Importantly, cells double mutant for Notch and the proneural genes form epidermis indicating that Notch acts only through the inhibition of proneural gene expression [16]. Thus, in this process Notch controls a binary fate decision (defined as a choice between two cell fates) between the epidermal and neural fates through the inhibition of a neural program. Hereafter, a mechanism where Notch resolves a binary fate choice through the inhibition of a specific program of differentiation will be referred to as lateral inhibition.

The second mechanism consists of binary fate decisions that specify distinct neural subtype identities. For instance, in the *Drosophila *peripheral nervous system (PNS), once the neural precursor (which is called SOP for Sensory organ precursor) has been specified via lateral inhibition, it divides in a stereotypical fashion to generate two intermediate progenitors (pIIb and pIIa) that communicate via Notch to establish their respective identities. These cells divide again to generate four cells, the identities of which are again established through communication between sister cells via Notch [17]. The description of Notch activity during specification of neural subtype identity has led to the binary switch model. Briefly, this model postulates that during fate choices between two subtype identities, Notch is both instructive for one fate and inhibitory for the other. This model is mainly based on the observation that during these fate decisions, the loss of Notch activity and the constitutive activity of the Notch pathway leads to opposite phenotypes. In addition, this model does not imply that Notch is instructive in an absolute manner but that it has an instructive capability in the context of a binary fate choice, meaning that it drives one specific fate from a bipotent progenitor.

Two important points need to be raised concerning the binary switch model. First, since Notch is able to trigger different outcomes depending on the cellular context, it is likely to cooperate with other influences during the specification of cell fate, which are missing in the simple model of the binary switch. Second, only the identification of Notch targets will enable us to ascertain that Notch has an instructive (rather than inhibitory) role in these fate decisions. Nevertheless, during both the selection of neural precursors and the specification of neural subtype identity, Notch appears to trigger a binary fate decision between two possible alternative identities. While in the first case the activity of Notch is strictly inhibitory, it is not yet clear whether the choices between two different neural subtype identities involve the inhibition of one fate or the promotion of the other fate or both activities as postulated in the binary switch model.

Divisions of the *Drosophila *SOPs exhibit stereotyped orientations and, consequently, stereotyped outcomes in terms of cell-type specification. Indeed, these outcomes are the result of a process of asymmetric segregation of cell fate determinants that relies on the stereotyped division orientation. At each of these divisions the Notch interactors Numb and Neuralized (Neur) are segregated to one of the two daughter cells. In turn, these molecules appear to bias the activity of the Notch pathway either negatively in the case of Numb or positively in the case of Neuralized, which potentiates the activity of Notch ligands [17]. Not all binary decisions regulated by Notch require asymmetric segregation of fate determinants, however. For instance, in the *Drosophila *eye the decision to adopt an R4 versus an R3 photoreceptor identity relies on Notch activity despite R3 and R4 not sharing a fixed lineage relationship (Figure 1D). In this system, the initial bias in Notch activity is provided by the activity of the Frizzled receptor (Fz) acting through Dishevelled (Dsh), which reinforces both *delta *and *neur *expression in the presumptive R3 cell [18-22].

Finally, Notch influences neural subtype specification in decisions that appear more complex than simple choices between two alternative fates. For instance, in the *Drosophila *eye disc, the loss of Notch activity induces a transformation of R7 photoreceptors into other subtypes of photoreceptors (R1 or R6), while conversely the constitutive activation of Notch in R1 and R6 photoreceptors can either force them to differentiate as R7 or less frequently as cone cells [23]. Thus, in this context, Notch activity cannot be restricted to a single R7 versus R1/6 binary fate decision. Further studies will be required to understand whether in this system Notch functions through a mechanism that does not involve binary fate decisions or whether it is in fact involved in several successive binary decisions.

Altogether, these studies suggest a role for Notch in binary fate decisions in two subsequent contexts: first, decisions between an epidermal and a neural fate, and second, during choices between two distinct neural subtypes. In addition, Notch specifies neural fates through decisions that appear more complex than simple binary fate choices.

## Notch activity in the vertebrate nervous system: an emerging role in cell fate diversification

**Figure 2:**
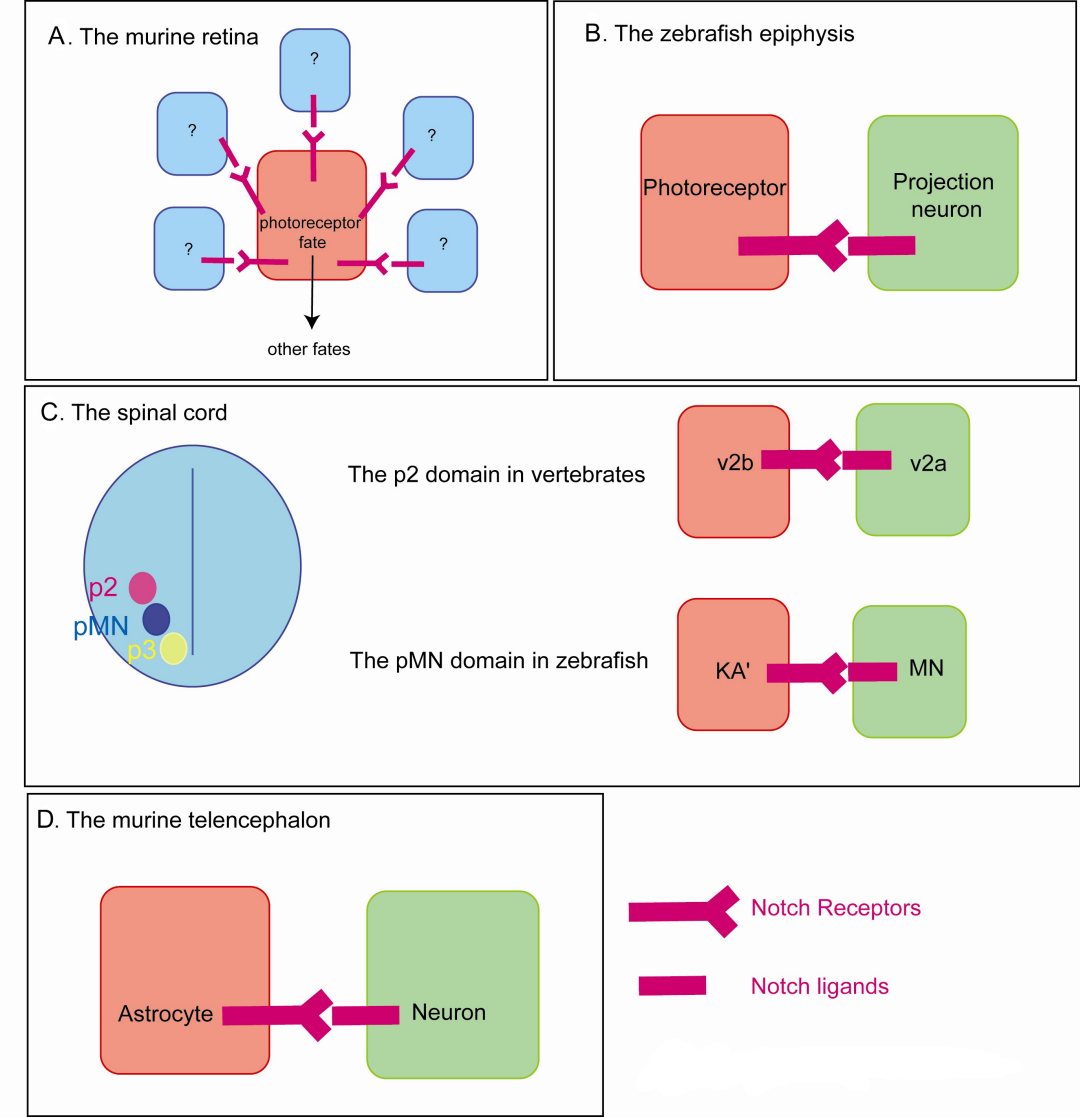
**A role for Notch during neural subtype specification in vertebrates**. Notch communication (in pink) influences cell fate in the murine retina, the zebrafish epiphysis, the vertebrate spinal cord as well as the murine telencephalon. **(A) **While in the murine retina the nature and number of signal-sending cells is unknown, Notch1 activity results in an inhibition of the photoreceptor fate, which would allow cells to adopt alternative fates. **(B) **In the zebrafish epiphysis, projection neuron precursors express high levels of Notch ligands, therefore inhibiting their neighbours from adopting a projection neuron fate. **(C) **A section of the vertebrate spinal cord is represented dorsal up with the three ventral progenitor domains: p2, pMN and p3. Whereas the Notch ligand Dll4 is expressed widely in p2 neural progenitors, only the future v2a cells are thought to retain its expression [34]. Note that in the case of the KA'/MN decision, it is not clear which are the ligand expressing cells, although one would expect an enrichment in the presumptive MN precursors [33]. **(D) **In the murine telencephalon, Notch communication helps in specifying the astrocytic fate together with the JAK/STAT pathway.

The Notch pathway is also a prominent signalling pathway in vertebrates with the additional complexity that it employs four different Notch receptors (Notch 1 to 4) and several different ligands (such as, for instance, Delta 1, 3 and 4 and Jagged 1 and 2 in mammals) compared to the single Notch receptor and the two ligands (Delta and Serrate) in *Drosophila *[1]. The Notch signalling pathway is involved in a process of selection of neural progenitors in the vertebrate neural tube [24]. However, as all neural tube cells eventually generate neural cells (either neurons or glia), Notch does not control the decision between epidermal versus neural fate as in *Drosophila*, but controls the timing of cell birth and differentiation. Notch prevents cells from differentiating, thus maintaining a pool of progenitors. Alterations in Notch activity also modify neuronal subtype specification. Given that the Notch pathway alters the timing of neurogenesis and that timing has been linked to cell fate [25], whether Notch acts directly during the specification of neuronal subtype identity in vertebrates has remained controversial for many years. Recent data obtained in the retina, spinal cord and epiphysis suggest, however, that in addition to its role in controlling the timing of neurogenesis, the Notch pathway directly controls the identity of a number of cell types. These effects are schematized in Figure 2.

In the rodent retina, ganglion cells, horizontal cells and cone photoreceptors are produced at early stages followed by amacrine and rod photoreceptors and finally bipolar cells and Muller glia at late stages [26]. Conditional inactivation of the *notch1 *gene induces the production of an excess of photoreceptors at the expense of other cell fates regardless of whether this inactivation is performed at an early or late stage. These data cannot simply be explained by a role for Notch in maintaining a pool of progenitors as one would expect an excess of bipolar or Müller cells (the latest fates) upon late inactivation of Notch1 if this were the case. Therefore, Notch appears to directly control cell fate through the inhibition of a photoreceptor identity [27,28]. Conversely, gain of Notch activity induces the production of an excess of Müller glia [29]. These data would argue for a photoreceptor/Müller glia switch. However, these cell types are not produced at the same time and the increased number of photoreceptors observed upon *Notch1 *inactivation occurs at the expense of all other cell types and not just Müller cells [26-28]. Hence, the phenotype observed upon loss of Notch1 might reflect a binary decision occurring between a photoreceptor and a cell that retains the potential for all other identities or various binary fate decisions between a photoreceptor and another differentiated cell (including a decision between a photoreceptor and a Müller cell) or even an effect that does not involve binary fate decisions at all (Figure 2A). Surprisingly, data obtained in other model systems do not support a role for Notch in inhibiting the photoreceptor fate. In the chick, knock down of *c-notch1 *using an antisense strategy leads to an excess of ganglion cells [30]. Moreover, in the zebrafish, inactivation of the Notch pathway slows down photoreceptor differentiation and severely impairs the production of Müller glial cells but does not result in an excess of photoreceptors [31]. Whether these different results reflect methodological differences, differences in the reduction of Notch activity or the employment of different strategies to specify cell fate in different species remains unclear.

The Notch pathway is also instrumental during the specification of cell fate in the ventral spinal cord (Figure 2C). In the pMN domain of the zebrafish spinal cord, GABAergic interneurons (KA') and primary motoneurons (MNs) are produced simultaneously, and in a number of cases are related by lineage - one KA' and one MN cell being generated from an asymmetric terminal division. In this context, constitutive activation of Notch promotes the specification of the KA' fate over the MN fate [32,33]. Similarly, Notch plays a role in specifying two distinct subtypes of interneurons: the v2a and the v2b from the p2 domain of the ventral spinal cord [34-36]. Interestingly, virtually all of these neurons are produced through a terminal v2a versus v2b decision [37], suggesting again a direct effect of Notch on fate specification. Finally, loss or gain of Notch activity affects cell fate within the zebrafish epiphysis, a dorsal diencephalic structure that contains two neuronal types: the photoreceptors and the projection neurons (Figure 2B). In this context as well, the Notch pathway directly controls the specification of cell fate as birth-dating experiments show that these two cell types are born simultaneously [38]. Altogether, these studies point towards a direct role for Notch during the specification of neuronal subtype identity in vertebrates. While most cases can reasonably be attributed to a role for Notch in binary fate decisions (KA'/MN, V2a/V2b, zebrafish epiphysis), it is possible that in systems such as the murine retina, Notch is involved in more complex decisions than single binary fate choices.

Another important question is whether the effects of the Notch pathway on specification of neuronal subtype identity are biased by asymmetric segregation of Notch interactors. The vertebrate retina is well-known for its apparent lack of fixed lineage relationships [39-41]. However, an elegant study by Poggi *et al*. [42] suggests a previously unanticipated logic in the retinal lineages. Indeed, theses authors describe the existence of a ganglion cell progenitor, labelled with a specific transgene, that divides with a specific orientation to generate one ganglion cell and one non-ganglion cell. This suggests that the vertebrate retina contains specific progenitors that can be identified molecularly and that produce a relatively stereotyped outcome. Whether such a progenitor exists for photoreceptors remains to be determined. Given the lineage data, asymmetric segregation of Notch interactors could occur in both KA'/MN and v2a/v2b contexts [32,33,37]. However, in the former case terminal divisions generating two KA' cells are also observed, suggesting that asymmetric segregation of Notch interactors is not an absolute requirement [32].

## Is Notch instructive in binary fate decisions?

In *Drosophila*, during the choice between a neural versus epidermal fate, Notch has always been described as inhibiting the neural fate rather than activating the epidermal fate. In contrast, during its later activity during the specification of neural subtype identity, it has been proposed to function as a binary switch, instructive for one fate and inhibitory for the other. However, in both the selection of a neural progenitor and neural specification, the cell has the choice between the 'A' fate and the 'B' fate, and whereas loss of Notch activity results in the production of two 'B' cells, constitutive activation of Notch signalling always leads to the production of two 'A' cells [15,17]. In the vertebrate spinal cord, constitutive activation of the Notch pathway is sufficient to induce the v2b fate at the expense of the v2a fate in the p2 domain [35], which led to the interpretation that Notch is instructive to specify this fate. Similarly, in the pMN domain constitutive activation of Notch forces cells towards the KA' fate at the expense of the MN fate [33], which led to the interpretation that Notch both instructs the KA' fate and inhibits the MN fate. It is important to note that while these interpretations appear reasonable and parsimonious, they have yet to be firmly established through the study of Notch target genes (see below).

The zebrafish epiphysis represents a slightly different case from the 'binary switch' model. Indeed, while the loss of Notch activity leads to an excess of projection neurons, under these conditions a significant proportion of cells are detected with a mixed photoreceptor/projection neuron identity. Furthermore, while forced activation of the pathway inhibits the expression of projection neuron markers, photoreceptor identity is not promoted. Therefore, in the epiphysis Notch does not function as a binary switch but serves to repress an undesired genetic programme in cells induced through a parallel mechanism to adopt a different fate [38]. In this context, Notch would not be instructive but would rather cooperate with another pathway that would be instructive for the activation of a photoreceptor fate.

Similarly, during the induction of the astrocytic fate in the murine telencephalon, Notch does not operate in a truly instructive manner but rather requires interaction with another signalling pathway. Neural progenitors of the mammalian brain produce neurons at early stages of gestation and glial cells later, which led to the hypothesis that recently born neurons could be responsible for providing astrocytic potential to their neighbouring neural progenitors at late stages of development. Coherent with this idea, young neurons express Notch ligands and overexpression of the constitutively active form of Notch is sufficient to trigger astrocytic differentiation in neural progenitors (Figure 2D). However, this effect is revealed only when the JAK/STAT (Janus kinase/Signal transducer and activator of transcription) pathway is activated. Moreover, Notch has been shown to regulate the competence to activate the JAK/STAT pathway through the demethylation of astrocyte-specific promoters. This demethylation is necessary for the induction of the astrocyte fate by the JAK/STAT pathway and is triggered by the nuclear factor I (NFI) A, which prevents binding of the methyltransferase DNMT1 to the astrocytic promoters [43]. Therefore, the expression of Notch ligands by young neurons appears to confer astrocytic potential to neighbouring neural progenitors but is not sufficient for specification of astrocytes. As such, the role of Notch in this context can be defined as permissive rather than instructive. Can the effect of Notch on astrocyte differentiation fit in the binary fate choice model? One could consider that the coordinated activities of the Notch and JAK/STAT pathways are responsible for a neural progenitor versus astrocyte fate decision. However, it will be important to understand whether telencephalic neural progenitors that have not activated the Notch pathway retain neurogenic potential. These data exemplify the fact that Notch communication can influence fate decisions by regulating the competence to respond to a second pathway without being instructive.

## Towards an understanding of the Notch-elicited response

To discover whether Notch really possesses the ability to induce specific neural fates, it is crucial to identify the molecular cascades acting downstream of Notch in the nervous system. During the selection of neural precursors, in both *Drosophila *and vertebrates, the intracellular domain of Notch together with its co-activator Suppressor of Hairless (Su(H)) elicits the transcriptional activation of the *Enhancer of Split *(*E'(Spl)*) family of bHLH transcriptional repressors. In turn, Enhancer of Split proteins repress the proneural genes [1,44-46]. This inhibition of proneural gene expression leads to the inhibition of a neuronal fate.

On the other hand, during the specification of neural subtypes in the *Drosophila *PNS, two pathways have been identified downstream of Notch. While one involves the canonical transcription factor Su(H) [47,48], the other involves the post-transcriptional inhibition of the RNA binding protein Mushashi. This post-transcriptional inhibition releases the inhibition of Mushashi (MSI) on Tramtrack69 (TTK69), a zinc finger transcription factor that functions as a repressor. Therefore, cells that have activated the Notch pathway show a more efficient translation of TTK69 [49,50]. Interestingly, the sheath/neuron cell fate decision does not seem to rely on either of these two mechanisms while the socket/hair decision relies on both *Su(H) *and *ttk69 *[47-50]. This suggests a complexity that is not fully understood even in the *Drosophila *PNS where these questions have been best studied. In addition, as Su(H) functions either as a negative regulator of transcription (through the recruitment of co-repressors such as histone deacetylases; see [2] for a review) or as an activator of transcription (when combined with Notch-intra and co-activators, including histone acetyl transferases; see [2] for a review), it is possible that some of the events that have been classified as Su(H)-independent in fact rely on Su(H) but show an equilibrium between the negative, Notch-independent effect and the positive Notch-dependent effect. Such an equilibrium has been observed during the regulation of *single-minded *(*sim*) gene expression in the *Drosophila *mesectoderm [51].

To understand whether Notch is instructive for the socket cell fate and/or inhibitory for the hair cell fate, it is crucial to identify the targets of *ttk69 *and *Su(H) *in the socket cells. A recent study shows that Notch and Su(H) together with their cofactor ventral veins lacking (vvl) activate *sox15*, a gene required for socket cell survival but not socket cell formation. Therefore, although Notch seems to be important to implement socket cell differentiation by activating *sox15*, it is not yet clear whether it is instructive for a socket fate. Part of the problem in answering this question could be that Notch regulates a plethora of socket differentiation genes rather than a single 'high level' regulator of socket fate. In contrast, both *Su(H) *and *sox15 *are required to prevent the expression of *D-pax2*, a regulator of hair cell differentiation. This last result suggests that an essential activity of Notch is to prevent the inappropriate activation of the hair cell program in the socket cell [52,53]. Even in *Drosophila*, it is not clear whether Notch specifies neural subtype identities through being instructive for specific fates, inhibitory for others or both in binary fate decisions. In the long run, we hope that approaches aiming at identifying targets for Su(H) and TTK69 will provide answers to this question.

Interestingly a target for Notch has been identified in a decision that appears more complex than a single binary fate decision in the *Drosophila *retina. Here, *D-pax2 *is required for proper cone cell differentiation [54]. Notch, *lozenge *and the epidermal growth factor (EGF) pathways are also all important for the formation of cone cells [55-57]. In addition, the combined activities of Notch, the EGF pathway and the transcription factor Lozenge are responsible for the specific expression of *D-pax2 *in the cone cells of the *Drosophila *eye disc through the direct binding of Su(H), Pointed (an EGF effector) and Lozenge to a *D-Pax2 *eye-specific enhancer [58]. Therefore, Flores *et al*. [58]proposed a model in which the [cells that would exhibit *lozenge *expression and both Notch and EGF activation at the same time would be fated to become cone cells. In the extended version of their model, the [combined activities of Notch, the EGF pathway and *lozenge *would allow for the generation of four distinct identities (undifferentiated cell, cone cell, R3, or R4 and R7). Interestingly, in this model, the R7 and the cone cells show the same level of Lozenge and EGF activity and only differ by their level of Notch activity; therefore, absence of Notch activation in R7 is thought to be responsible for the absence of *D-pax2 *expression in the R7 photoreceptor. However, a study by Cooper and Bray [23] contradicts this model as it shows both activation of the Notch pathway in the R7 cells as well as a prominent role for Notch on the specification of the R7 photoreceptor fate. Nevertheless the data obtained by Flores *et al*. [58] nicely shows that Notch can be instructive for specifying cell fate in the nervous system, although it requires cooperation with other influences Further studies will be necessary to understand what controls the context-dependent effect of Notch that functions as an inhibitor of *D-pax2 *in the PNS and as an activator in the retina.

How is Notch activity transduced in contexts where it directly regulates fate specification in vertebrates? In the zebrafish spinal cord, the KA'/MN fate decision relies on the canonical Su(H)-dependent pathway [33]. In order to understand whether, in this context, Notch acts through the activation of the KA' fate, the inhibition of the MN fate or both, it will be important to identify the targets of a Su(H)-Notch-intra complex and analyze their activities. While no KA' fate inducers are available to date (nor KA' differentiation genes), one can speculate that the inhibition of the motoneuron fate by Notch activity could lead to the down-regulation of the proneural gene *olig2*, which is both necessary and sufficient for the specification of primary motoneurons in zebrafish [59]. Similarly, inhibition of the v2a fate by Notch could involve the down-regulation of *lhx3 *and *chx10 *expression while the three transcription factors Scl, Gata2 and Gata3 could mediate the effects of Notch on the activation of the v2b fate in the spinal cord [34,35]. Before the molecular mechanisms that transduce the Notch effect in this v2a/v2b fate decision have been elucidated, it is not possible to conclude whether Notch is instructive for the v2b fate.

Much work thus remains to be done before we understand the molecular pathways by which Notch influences cell fate in these different contexts and we establish whether Notch acts through the inhibition of specific fates, whether it is inductive for others or whether it influences the response to other signalling pathways.

## Alternative models for the role of Notch in binary fate decisions

**Figure 3:**
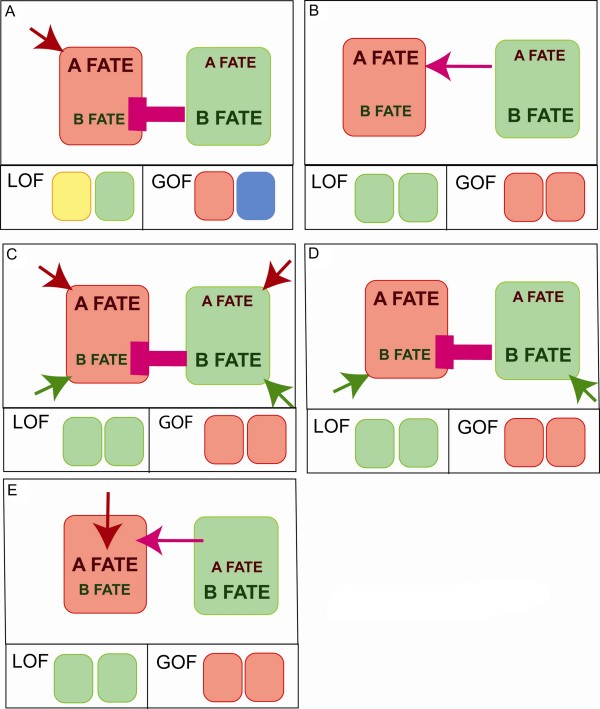
**Models for Notch activity in binary decisions**. Cells have the choice between two subtype identities, the A fate (in red) and B fate (in green), which can be induced by an A-inducing signal (in red) or a B-inducing signal (in green). **(A-D) **Notch activity (in pink) influences cell fate either by inhibiting the B fate (A, B, D) or by activating the A fate (C). In the epiphysis (A), Loss of Notch activity (LOF) results in the production of AB cells (yellow) while gain of Notch activity (GOF) results in the production of neurons with no identity (blue). **(E) **Notch regulates the competence to respond to a second signalling pathway.

The results obtained upon gain or loss of Notch activity in the epiphysis significantly differ from the binary switch model in two ways: first, loss of Notch results in the production of cells with a double-identity; and second, Notch is not instructive for the alternative (photoreceptor) fate. These data have been interpreted to mean that Notch acts to inhibit the projection neuron fate (the B fate in Figure 3A) whereas the photoreceptor fate (A fate) is promoted by a yet to be discovered inducing signal. In contrast, in the binary switch model, Notch is thought to instruct the A fate (Figure 3B) and possibly inhibit the B fate in parallel. Since it is not possible at this point to conclude whether Notch really possesses instructive activity in binary fate decisions made in the nervous system (see above), we propose two alternative models to the binary switch in which Notch activity is mainly inhibitory with respect to one fate. In the first scenario (Figure 3C), Notch only has a repressive activity on the acquisition of the B fate and this activity of Notch allows cells to choose between the influence of two inductive signals, one for the A fate and one for the B fate. In a variation of this scenario, the A fate would be a natural 'default state' that would not require the intervention of a signal (Figure 3D). In these two cases, Notch activity is only repressive, as in the epiphysis (Figure 3A). However, in contrast to the epiphysis, the B cells are initially able to become A cells, either because they are exposed to or can respond to the A fate-inducing signal or because this A fate is a 'default state'. These scenarios, however, do not explain that mixed identity cells have not been observed in binary switch paradigms such as the *Drosophila *PNS. The most trivial explanation for this would be that in other binary decisions, the presence of cells with a mixed identity has been missed due to the lack of appropriate markers. Alternatively, these systems could show no tolerance for the simultaneous activation of two genetic programs, either because the B genetic program would have the capacity to override the A program or because AB cells would die. The data obtained in the murine telencephalon [43] suggest a fifth model (Figure 3E) where Notch regulates the competence to respond to another signalling pathway thereby allowing for the induction of the A fate without being instructive for it. Interestingly, a similar model was first proposed by Cooper and Bray [23] to explain the cooperation between the Notch and the Ras pathways during the specification of the R7 identity in the *Drosophila *eye disc. Therefore, apart from the binary switch-instructive model, it is possible to explain the effects obtained in binary fate decisions with simple alternative models involving either an inhibitory effect of Notch on specific fates or the regulation of the competence to respond to other signalling pathways. Further studies will clarify how cooperation with other pathways impacts on Notch-driven binary fate choices.

An interesting aspect of these models is that they might help understanding of how Notch activity triggers different effects in different cell contexts. Indeed, in the third and fifth models (Figure 3C, E) Notch cooperates with an additional pathway during the specification of cell fate. Therefore, combination of Notch pathway activation with different combinations of other signalling pathways would allow different outcomes and help diversify the response to Notch activation. Along the same line, in the fourth model, the cell that has received Notch activation and is therefore inhibited from producing a B type of cell, differentiates as an A-type of cell as a 'default fate', meaning that it adopts this fate as the result of the specific combination of transcription factors and chromatin marks found in this particular cell in the absence of additional signalling activity. Here again, activation of Notch in different types of cells (expressing different combinations of transcription factors and chromatin marks) would be expected to produce different outcomes.

## A new way to generate cell fate diversity?

The mechanism of Notch-driven fate specification in the epiphysis could represent an attractive 'economical' way of generating fate diversity, although it remains to be understood whether it can also occur in other cellular contexts. Indeed, using only two signals (here one inductive and one inhibitory, although, in theory, the same outcome could be obtained using two different inductive signals) three cell fates can be produced: an A fate (which receives both signals), a B fate (which receives none) and a mixed AB fate (which receives only the inductive fate). However, in the epiphysis, the mixed identity cells that are frequently detected upon loss of Notch activity are only rarely observed in the wild-type epiphysis during development [38] and are very unlikely, therefore, to represent a true cell fate. In contrast, the intrinsically photosensitive retinal ganglion cells (ipRGCs) of the mammalian retina express the characteristics of both projection neurons and photoreceptors as they express the melanopsin photopigment and are photosensitive [60-62]. Thus, ipRGCs represent a potential example of a mixed 'AB' fate. In this system, ganglion cells were originally photosensitive and would have lost this property during evolution, except in ipRGCs [63]. Such an evolutionary scenario requires a mechanism for the segregation of the photoreceptive and the projection neuron functions. It would be interesting to test whether constitutive activation of Notch could force ipRGCs to resolve their mixed identity. Arendt [63] actually proposes that segregation of function is a general evolutionary driving force for expanding cellular diversification. The results obtained in the zebrafish epiphysis suggest that the inhibitory activity of Notch on specific genetic programs could have contributed to such a segregation, therefore contributing to an expansion of neuronal subtype diversity.

## Conclusion

Although binary fate decisions are most likely an oversimplified way of looking at Notch activity, as Notch controls many other processes, including fate decisions between more than simply two fates, they represent a convenient framework to compare Notch activity in different systems. In addition, while the binary fate decisions triggered by Notch have been initially categorized in two general classes - lateral inhibition and 'binary switch', which were thought to correspond to initial decisions to adopt a neural fate and the choice of a neural subtype identity, respectively - the situation appears to be more complex than this simple dichotomy. Indeed, in the epiphysis the specification of neural subtype identity involves the inhibition of a specific genetic program (lateral inhibition) rather than a 'switch activity'. In this context, as well as during the specification of astrocytes in the murine telencephalon, Notch does not appear as the 'magic fate switcher' but rather acts cooperatively with other pathways during the specification of cell fate. Also surprising is the observation that whereas Notch activity is implicated in an increasing number of fate decisions (including recently in vertebrate neuronal subtype specification), very little is known concerning its activity as an inhibitory or instructive signal on specific genetic programs leading to fate choice. We believe that future work devoted to the understanding of the cascade of events following Notch activation will pave the way for a better understanding of its action as well as elucidate its potential evolutionary significance.

## Abbreviations

bHLH: basic helix-loop-helix; CNS: central nervous system; EGF: epidermal growth factor; ipRGC: intrinsically photosensitive retinal ganglion cell; PNS: peripheral nervous system; JAK/STAT: Janus kinase/Signal transducer and activator of transcription; SOP: Sensory organ precursor; Su(H): Suppressor of Hairless; TTK69: Tramtrack69.

## Competing interests

The authors declare that they have no competing interests.

## Authors' contributions

EC wrote the paper and PB revised the manuscript.
